# Flexible Laryngoscopy in Unexplained Cough: Institutional Outcomes and Community Practice Patterns

**DOI:** 10.7759/cureus.116687

**Published:** 2026-09-21

**Authors:** Nader Wehbi, Kira Wang, Helena Yip

**Affiliations:** 1 Otolaryngology - Head and Neck Surgery, University of Arizona College of Medicine - Phoenix, Phoenix, USA; 2 Chemistry, Yale University, New Haven, USA; 3 Otolaryngology - Head and Neck Surgery, University of Arizona College of Medicine – Tucson, Tucson, USA

**Keywords:** chronic cough management, flexible laryngoscopy, neurogenic cough, practice patterns, proton pump inhibitor

## Abstract

Background

Flexible laryngoscopy is routinely performed by otolaryngologists for unexplained chronic cough. The objectives were twofold: first, to ascertain institutional outcomes and, second, to understand community provider practice patterns to further inform its role in evaluation.

Methodology

This retrospective case series and cross-sectional survey was conducted at a single tertiary-care academic institution (2018-2024) and a regional otolaryngology continuing medical education conference (February 2025). For the retrospective case series, the study included adult patients seen at the University of Arizona’s tertiary laryngology center for unexplained chronic cough from 2018 to 2024. Patients met the inclusion criteria if cough persisted for >8 weeks and they either had not responded to prior proton pump inhibitor (PPI) and asthma therapy or had gastroesophageal reflux disease and asthma ruled out by specialists. The senior author routinely performed flexible laryngoscopy for all patients presenting with unexplained chronic cough. Normal and abnormal laryngeal findings in this retrospective study simply referred to the presence or absence of a mass, ulceration, or paralysis. For the cross-sectional survey, optional anonymous paper surveys were distributed to otolaryngology providers attending a regional otolaryngology continuing medical education conference in Tucson, Arizona, in February 2025.

Results

In our retrospective case series, 53 of 56 (94.6%) patients had normal laryngoscopic exams in their initial evaluation for unexplained chronic cough. Three (5.4%) patients had abnormalities on laryngoscopy, which included paradoxical vocal fold movement (3.6%, N = 2) and known vocal fold paralysis (1.8%, N = 1). Within our cross-sectional survey of 55 providers, 43 (78.2%) reported frequently performing laryngoscopy. Half of providers (49.1%, N = 27) indicated that findings rarely changed treatment. Common laryngoscopy findings included arytenoid erythema/edema (54.5%, N = 30) or normal exams (41.8%, N = 23). PPIs were the most frequent first-line therapy initiated (69.1%, N = 38).

Conclusions

In this single-institution small case series, laryngoscopy findings in unexplained chronic cough were predominantly normal or benign, with no malignancies identified. These findings suggest that patients with unexplained chronic cough that significant laryngeal pathology may be uncommon, while still supporting the importance of direct visualization before diagnosing unexplained cough. In this limited cross-sectional survey, flexible laryngoscopy was not universally adopted among community providers, highlighting variability in the evaluation for chronic cough. Empiric PPI therapy remains frequently prescribed despite guideline recommendations favoring neuromodulators and cough suppression therapy.

## Introduction

Chronic cough accounts for 11% of primary care visits and remains a prevalent, vexing condition, especially when refractory to standard treatments [1]. Patients with an unexplained chronic cough are often referred to an otolaryngologist for further evaluation. The usual workup includes an in-office flexible laryngoscopy. Flexible laryngoscopy is useful to assess laryngeal anatomy, identify structural lesions, and evaluate voice and airway complaints. Its value is well established in the workup of hoarseness, dysphagia, stridor, and suspected malignancy, where visualization of the larynx often directly informs diagnosis and management [2]. In patients who present with cough in combination with voice changes or swallowing difficulty, laryngoscopy can help identify vocal fold lesions, vocal fold paresis, laryngopharyngeal reflux, or obstructive pathology.

Neurogenic cough is the leading explanation for many cases of unexplained chronic cough, characterized as a laryngeal sensory neuropathic disorder leading to hypersensitivity of the cough reflex [3-5]. It is a diagnosis of exclusion, only made after more common causes of cough have been ruled out. Because it arises from a functional hypersensitivity rather than a structural abnormality, laryngoscopic findings in these patients are often normal or show only minor, nonspecific abnormalities. As early as 1908, historical otolaryngology texts recognize that laryngoscopy findings in patients with chronic cough are largely normal [6].

Although laryngeal cancer most often presents with hoarseness or dysphagia, it can, in some rare cases, manifest initially as a persistent cough [7]. Laryngoscopy therefore remains valuable in the evaluation of chronic cough by helping exclude structural or malignant disease before symptoms are attributed to neurogenic etiologies.

This study describes laryngoscopy findings in patients with unexplained chronic cough at a single institution and incorporates a provider survey to better understand current community practice patterns in evaluation and management.

## Materials and methods

The study consists of two parts. The first is a retrospective review from 2018 to 2024 of the author’s own series of patients with unexplained cough. The second part is a cross-sectional survey of otolaryngology providers at a regional continuing medical education (CME) course.

Institutional cohort review

Following institutional review board approval, we conducted a retrospective review of medical records for adult patients evaluated for unexplained cough at our institution. Inclusion criteria required patients to be over 18 years of age with an unexplained chronic cough persisting longer than eight weeks that remained unexplained despite evaluation and exclusion of common etiologies. Eligible patients must have either failed prior treatment with proton pump inhibitors (PPIs) and asthma therapies or had undergone workups that ruled out gastroesophageal reflux disease (GERD) and asthma as contributing causes. Patients must also have been evaluated with flexible laryngoscopy. The senior author was routinely performing laryngoscopy for all patients presenting with unexplained chronic cough. Flexible laryngoscopy documentation would specifically note normal or abnormal findings of the base of the tongue, vallecula, larynx, piriform sinuses, and epiglottis. Normal and abnormal laryngeal findings in this study simply referred to the presence or absence of a mass, ulceration, or paralysis. As paresis as well as paradoxical vocal fold movement can be a subjective finding and may differ depending on the evaluator, it was not included in the parameters measured. The videolaryngoscopy was recorded and archived. Blinded review of whether a laryngeal mass was present was not deemed necessary. Videostroboscopy was not performed as these patients did not come in for voice complaints, but just the cough. Along the same vein, they did not present with symptoms suggestive of inducible laryngeal obstruction, and therefore provocative maneuvers were not performed. Often, the laryngoscopy was poorly tolerated and would not allow time to perform both static and dynamic evaluations. These patients often have significant gagging and laryngeal adduction with laryngoscopy, thus making provocative maneuvers unnecessary. Patients with concomitant symptoms such as hoarseness, dysphagia, or odynophagia were excluded from analysis (Table 1).

**Table 1:** Inclusion and exclusion criteria. PPI = proton pump inhibitor; GERD = gastroesophageal reflux disease

Inclusion	Exclusion
Age >18 years	Hoarseness
Chronic cough persisting >8 weeks	Dysphagia
Isolated chronic cough without concomitant hoarseness, dysphagia, or odynophagia	Odynophagia
Cough remained unexplained despite evaluation and exclusion of common etiologies	
Failed prior treatment with PPIs and asthma therapies, or GERD and asthma were ruled out	
Flexible laryngoscopy was performed as part of the evaluation	

Data collection included patient demographics, diagnostic evaluations, laryngoscopy findings, initiated therapies, and outcomes.

Survey design and distribution

A cross-sectional survey was distributed to assess otolaryngology provider practices and perceptions regarding the utility of laryngoscopy in the evaluation of patients with isolated chronic cough. The survey consisted of 10 multiple-choice questions covering provider demographics (e.g., training level, years in practice, and subspecialty orientation), frequency of chronic cough encounters per week, diagnostic use of laryngoscopy for chronic cough, perceived diagnostic yield, most common laryngoscopic findings, and preferred first-line treatments. Providers were also asked to estimate how often laryngoscopy findings influenced their management decisions.

The survey was distributed to otolaryngology providers, including MD/DO physicians, nurse practitioners, and physician assistants, during a regional CME conference held in Tucson, Arizona, on February 8, 2025. Participation was voluntary and anonymous. Providers were instructed to complete the survey based on their routine clinical practice and recent experiences managing patients with chronic cough. There were 122 registered attendees, and 75 were MD/DOs and nurse practitioners/physician assistants. The rest were medical students, registered nurses, and vendors. The paper surveys were left on the conference tables in front of each seat. An announcement was made at the start of the laryngology session of the conference asking attendees who would qualify as otolaryngology providers to fill out the surveys. Their completion of the survey was completely voluntary. There was no method to prevent duplication. There were no incomplete surveys. The completed surveys were then collected from the conference tables at the end of the half-day session. The attendees for different sessions varied, and therefore it was hard to ascertain the response rate. For the purposes of the survey, “chronic cough” was used to refer to unexplained chronic cough. This terminology was used to reflect language commonly encountered in clinical practice, where patients may present with chronic cough before an unexplained diagnosis has been formally assigned.

Statistical analysis

Survey responses were analyzed for the 53 providers who answered both the most common laryngoscopy finding question and the first-line treatment question. Providers reporting a normal examination were compared directly with providers reporting arytenoid erythema/edema rather than with the overall cohort. Categorical comparisons were made using Fisher’s exact test given the small cell counts and are reported with risk differences, risk ratios, and odds ratios (ORs) with 95% confidence intervals (CIs). First-line treatment was a single-choice question, so the PPI and neuromodulator comparisons are not independent, and no correction for multiple testing was applied. Exploratory logistic regression was then used to test whether laryngoscopy finding, years in practice (15+ years versus less than 15 years), or practice type predicted PPI selection, using likelihood ratio tests and profile likelihood CIs. Firth penalized regression was used to confirm these estimates given the small cell counts. Analyses were performed in Python 3.10.

## Results

Part 1: Retrospective review

Patient Demographics

A total of 56 patients with unexplained chronic cough were included in our cohort review. The mean age was 64.6 years (SD = 14.8), and 37 (66%) patients were female. The average symptom duration before presentation was 64.4 months (SD = 78.2). All patients had previously received PPIs and asthma therapies or had undergone evaluation for GERD and asthma without clinical improvement. Reported risk factors included a history of smoking (21.4%, N = 12) and a preceding viral upper respiratory tract infection before symptom onset (16.1%, N = 9).

Laryngoscopy Findings

Flexible laryngoscopy showed normal findings of the base of the tongue, vallecula, larynx, piriform sinuses, and epiglottis in 53 out of 56 (94.6%) patients. Of the remaining three (5.4%) patients with abnormal findings, one was found to have right true vocal cord paralysis, likely secondary to comorbid lung cancer. The remaining two patients were found to have paradoxical vocal cord movement. Despite these abnormal findings, the management approach remained unchanged, consisting of gabapentin initiation and referral for behavioral cough suppression therapy.

Part 2: Survey findings

A total of 53 otolaryngology providers completed our cross-sectional survey, the results of which are presented in Table 2.

**Table 2:** Unexplained chronic cough laryngoscopy survey and results.

Survey question	Answer choices	Response (% of respondents, N = 53)
1. I’m a:	a. MD/DO	a. 72.7%, N = 40
	b. Nurse Practitioner	b. 16.4%, N = 9
	c. Physician Assistant	c. 5.5%, N = 3
	d. Other	d. 5.5%, N = 3
2. Length of practice:	a. In training	a. 5.5%, N = 3
	b. 0–5 years	b. 16.4%, N = 9
	c. 5–10 years	c. 7.3%, N = 4
	d. 10–15 years	d. 10.9%, N = 6
	e. 15+ years	e. 60.0%, N = 33
3. My practice is:	a. Comprehensive ENT	a. 85.5%, N = 47
	b. Subspecialty-oriented ENT	b. 10.9%, N = 6
	c. No answer	c. 3.6%, N = 2
4. I see chronic cough in a week:	a. Almost never	a. 1.8%, N = 1
	b. Occasionally	b. 12.7%, N = 7
	c. Sometimes	c. 25.5%, N = 14
	d. Frequently	d. 41.8%, N = 23
	e. Almost always	e. 18.2%, N = 10
5. Patients referred to me for chronic cough usually have tried and failed treatments for (circle all that apply):	a. Asthma	a. 67.3%, N = 37
	b. GERD	b. 72.7%, N = 40
	c. Allergies	c. 80.0%, N = 44
	d. Other ____________	d. 9.1%, N = 5
6. Do you perform diagnostic laryngoscopy on patients with isolated chronic cough and no other symptoms (e.g., hoarseness, dysphagia)?	a. Almost never	a. 1.8%, N = 1
	b. Occasionally	b. 5.5%, N = 3
	c. Sometimes	c. 10.9%, N = 6
	d. Frequently	d. 18.2%, N = 10
	e. Almost always	e. 60.0%, N = 33
	f. No answer	f. 3.6%, N = 2
7. Of the chronic cough patients you perform laryngoscopy on, what proportion have positive findings?	a. 0–10%	a. 23.6%, N = 13
	b. 10–30%	b. 25.5%, N = 14
	c. 30–50%	c. 25.5%, N = 14
	d. 50–75%	d. 9.1%, N = 5
	e. >75%	e. 10.9%, N = 6
	f. No answer	f. 5.5%, N = 3
8. For the chronic cough patients you perform laryngoscopy on, what is the most common finding?	a. Arytenoid erythema/edema	a. 54.5%, N =30
	b. Vocal fold mass	b. 0.0%, N = 0
	c. Vocal fold ulceration	c. 0.0%, N = 0
	d. Motion impairment	d. 0.0%, N = 0
	e. Normal exam	e. 41.8%, N = 23
	f. No answer	f. 3.6%, N = 2
9. For patients with unexplained chronic cough and no other symptoms, what is your first-line treatment?	a. Proton pump inhibitors	a. 69.1%, N = 38
	b. Neuromodulators	b. 18.2%, N = 10
	c. Antibiotics	c. 0.0%, N = 0
	d. Prednisone taper	d. 9.1%, N = 5
	e. Low dose opioids	e. 1.8%, N = 1
	f. No answer	f. 1.8%, N = 1
10. How often do scope findings change your plan of treatment for the chronic cough?	a. Almost never	a. 20.0%, N = 11
	b. Occasionally	b. 29.1%, N = 16
	c. Sometimes	c. 32.7%, N = 18
	d. Frequently	d. 14.5%, N = 8
	e. Almost always	e. 1.8%, N = 1
	f. No answer	f. 1.8%, N = 1

Provider Demographics and Practice Characteristics

The majority of survey takers were physicians (72.7% MD/DO, N = 40), followed by nurse practitioners (16.4%, N = 9) and physician assistants (5.5%, N = 3). Three survey respondents identified their profession as “other” (5.5%, N = 3). Years in practice were skewed toward senior clinicians: 60.0% (N = 33) had been in practice for over 15 years, 10.9% (N = 6) for 10-15 years, 7.3% (N = 4) for 5-10 years, 16.4% (N = 9) for 0-5 years, and 5.5% (N = 3) were still in training. Most providers (85.5%, N = 47) worked in comprehensive otolaryngology settings, while 10.9% (N = 6) practiced in subspecialty-oriented otolaryngology, and 3.6% (N = 2) did not answer. In terms of caseload, chronic cough was seen each week “almost always” by 18.2% of providers (N = 10), “frequently” by 41.8% (N = 23), “sometimes” by 25.5% (N = 14), “occasionally” by 12.7% (N = 7), and “almost never” by 1.8% (N = 1). Patients referred to these providers for chronic cough typically failed treatments for allergies (80.0%, N = 44), GERD (72.7%, N = 40), and asthma (67.3%, N = 37), with 9.1% (N = 5) indicating other therapies such as antibiotics, mental health counseling, and chronic obstructive pulmonary disease therapy.

Diagnostic Laryngoscopy Utilization and Outcomes

When evaluating patients with unexplained chronic cough, 78.2% of providers (N = 43) reported performing diagnostic laryngoscopy either “almost always” or “frequently.” Laryngoscopy findings influenced treatment plans “almost never” for 20.0% of providers (N = 11), “occasionally” for 29.1% (N = 16), “sometimes” for 32.7% (N = 18), “frequently” for 14.5% (N = 8), and “almost always” for 1.8% (N = 1). These findings are illustrated in Figure 1.

**Figure 1:**
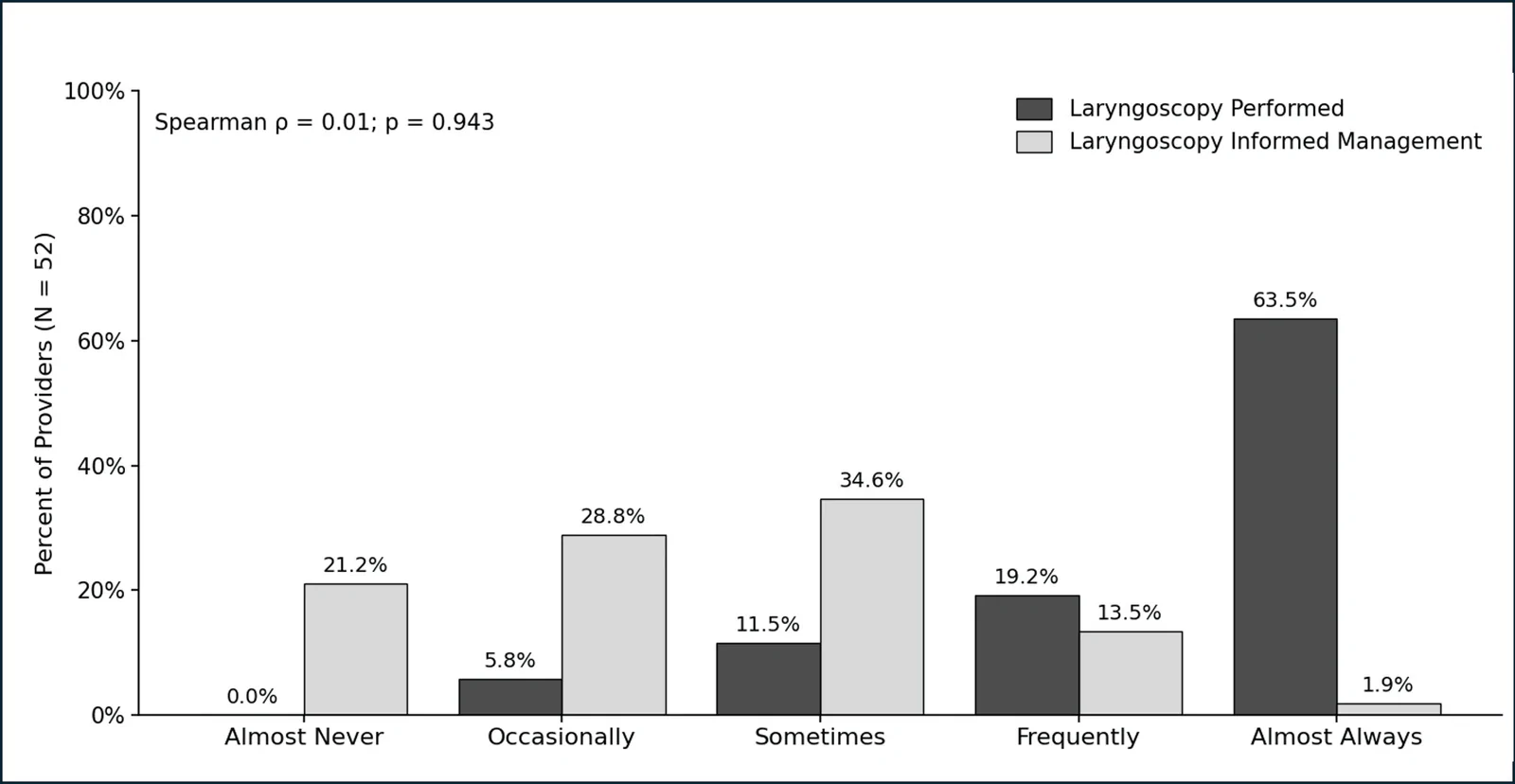
Laryngoscopy in unexplained chronic cough.

Positive findings on laryngoscopy were reported to be found in less than half of isolated chronic cough patients by 74.6% of providers (N = 41). Specifically, 23.6% of providers (N = 13) observed abnormalities in 10% or fewer of patients, 25.5% (N = 14) found abnormalities in 10-30%, and another 25.5% (N = 14) found abnormalities in 30-50% of patients. Only 20% of providers (N = 11) reported finding abnormalities in a majority of their patients, with 9.1% (N = 5) seeing them in 50-75% and 10.9% (N = 6) in >75%. When asked to identify the most common laryngoscopy finding in their practice, only two answer choices were chosen among all providers. Overall, 54.5% of providers (N = 30) selected arytenoid erythema or edema, while 41.8% (N = 23) reported a normal examination as the most frequent result.

In our survey, PPIs were the most frequently prescribed first-line treatment for unexplained chronic cough by the majority of providers (69.1%, N = 38), followed by neuromodulators (18.2%, N = 10), prednisone tapers (9.1%, N = 5), and low-dose opioids (1.8%, N = 1); antibiotics were not selected. Even among the 23 providers who reported “normal” as the most common laryngoscopy finding, 11 (47.8%) still selected PPIs as first-line therapy, and only eight (34.8%) selected neuromodulators. Of the 30 providers reporting arytenoid erythema/edema as the most common laryngoscopy finding, 27 (90.0%) chose PPIs as first-line therapy and two (6.7%) chose neuromodulators. These results are illustrated in Figure 2.

**Figure 2:**
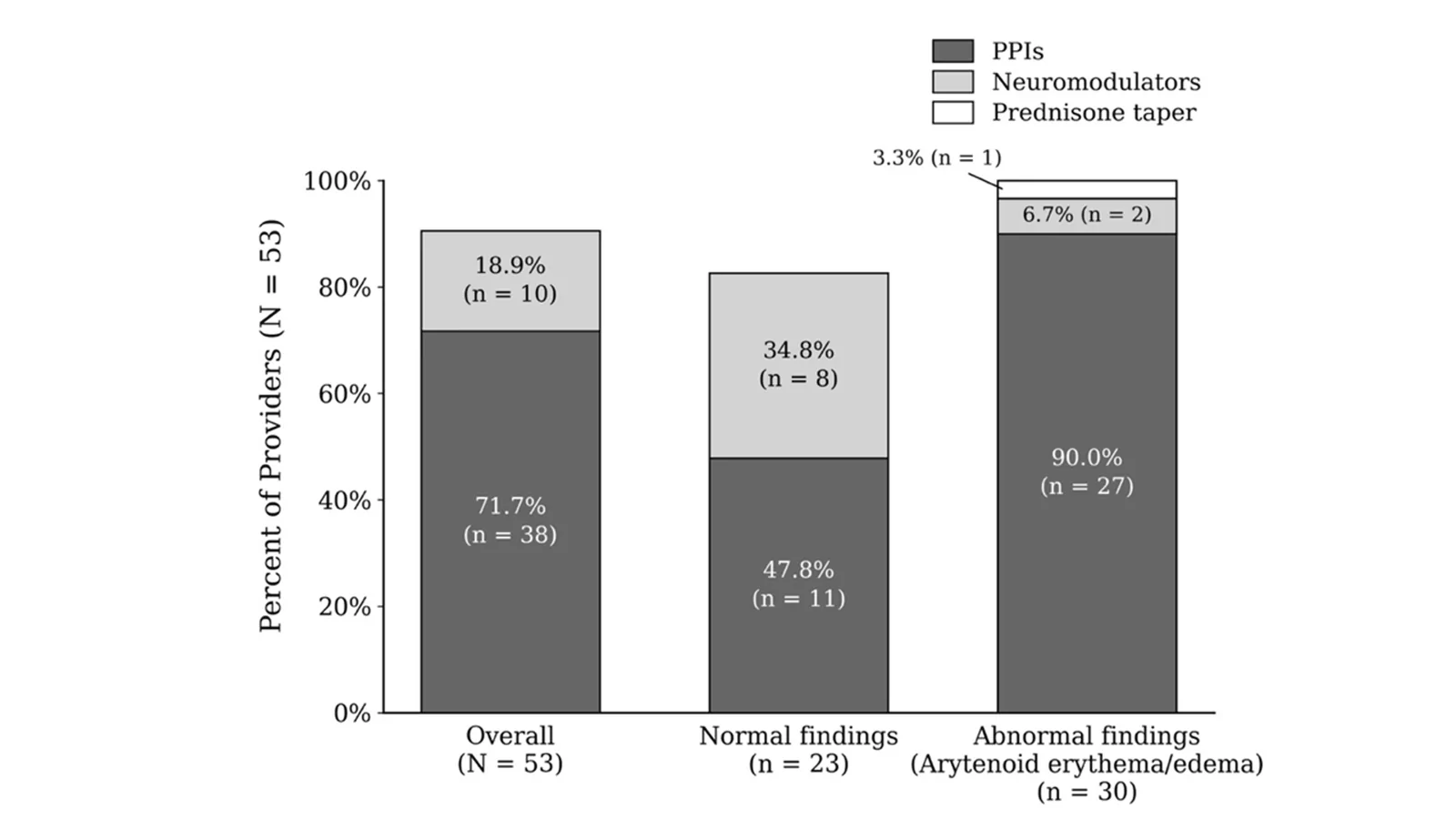
First-line therapy stratified by laryngoscopy findings. PPI = proton pump inhibitor

A total of 55 otolaryngology providers completed our cross-sectional survey. Providers who reported arytenoid erythema/edema as their most common laryngoscopy finding selected PPIs as first-line therapy more often than providers who reported a normal examination (90.0%, N = 27/30 vs. 47.8%, N = 11/23; p = 0.002), a difference of 42.2 percentage points (95% CI = 17.4 to 61.9) with an OR of 9.3 (95% CI = 2.0 to 61.7). The opposite was true for neuromodulators, which were selected by 6.7% (N = 2/30) of providers reporting arytenoid erythema/edema and 34.8% (N = 8/23) of providers reporting a normal examination (p = 0.014), a difference of 28.1 percentage points (95% CI = 6.4 to 49.0) with an OR of 0.13 (95% CI = 0.01 to 0.82). In exploratory logistic regression, the association between laryngoscopy finding and PPI selection persisted after adjusting for years in practice and practice type (adjusted OR = 10.9, 95% CI = 2.7 to 57.8). Neither years in practice (15+ years vs. less than 15 years: OR = 1.06, 95% CI = 0.24 to 4.40) nor subspecialty practice (OR = 1.39, 95% CI = 0.17 to 13.3) predicted PPI selection.

## Discussion

Unexplained chronic cough is a debilitating condition that is often difficult to treat. Evaluation requires otolaryngologic assessment to rule out structural laryngeal pathology before assigning an unexplained etiology. Although chronic cough alone rarely represents the initial presentation of laryngeal malignancy, direct visualization remains important [7-9]. This concern is greater in patients with prolonged hoarseness, for which current guidelines recommend referral to an otolaryngologist when symptoms persist for three weeks or longer [10]. In our series, laryngoscopy was normal in 94.6% of patients (53 of 56), identified no laryngeal malignancy, and did not alter treatment. These findings are corroborated by prior literature [11,12].

Our provider survey found that approximately 78.2% of community otolaryngology providers (43 of 55) reported performing laryngoscopy “almost always” or “frequently” when evaluating patients with unexplained chronic cough. This rate is lower than expected, as laryngoscopy should be considered part of an evaluation before attributing chronic cough to unexplained chronic cough, which is a diagnosis of exclusion. Direct visualization provides an important safeguard against missing structural laryngeal disease, including rare malignancy, before assigning a diagnosis of unexplained chronic cough.

Half of the surveyed providers indicated that laryngoscopy findings “almost never” or only “occasionally” influenced their treatment decisions, while 32.7% (18 of 55) reported that findings “sometimes” affected management. Only two laryngoscopy findings were reported across all respondents, which were a normal examination and arytenoid erythema or edema. Notably, approximately half (47.8%, 11 of 23) of providers reported prescribing PPIs as first-line treatment even in the setting of a normal laryngoscopic examination.

Our findings underscore a discrepancy between guideline-based recommendations and current clinical practice in the management of neurogenic cough. The American College of Chest Physicians (ACCP) guidelines recommend that, in cases of unexplained chronic cough, neuromodulators and behavioral therapy should be considered first-line treatments. They specifically advise against the empiric use of PPIs in the absence of typical gastroesophageal reflux symptoms or objective evidence of reflux [13]. Clinical trials have consistently shown that PPIs offer no significant benefit for chronic cough unless there is documented reflux [14-16]. In contrast, randomized studies have demonstrated that neuromodulatory agents, such as gabapentin and amitriptyline, and cough-suppression therapy can lead to meaningful improvements in both cough frequency and quality of life [13,17-19].

This study is limited by its modest provider sample size and regional sampling, which may not fully represent national practice patterns. The survey relied on self-reported data, introducing potential recall and response biases. The survey questions on first-line treatment options were not inclusive and did not include behavioral therapy. Additionally, the cross-sectional design precludes assessment of causality or temporal trends in provider behavior. In addition, the survey reflects reported practice rather than directly observed clinical behavior. Limitations in the retrospective chart review include a single-center retrospective design, potential selection and referral bias, and the possibility that the senior author’s routine performance of laryngoscopy on all chronic cough patients limits generalizability. The cohort definition of unexplained chronic cough may introduce diagnostic selection bias because it is a diagnosis of exclusion. Future surveys should incorporate current trends in multimodal and escalation of treatments for chronic cough. A large multi-institutional study on laryngoscopy findings in patients with unexplained chronic cough could also further elucidate the incidence of laryngeal pathology in this cohort of patients.

## Conclusions

In this single-institution small case series, laryngoscopy findings in unexplained chronic cough were predominantly normal or benign, with no malignancies identified. These findings suggest that patients with unexplained chronic cough that significant laryngeal pathology may be uncommon, while still supporting the importance of direct visualization before diagnosing unexplained cough. In this limited cross-sectional survey, flexible laryngoscopy was not universally adopted among community providers, highlighting variability in the evaluation for chronic cough. Empiric PPI therapy remains frequently prescribed despite guideline recommendations favoring neuromodulators and cough-suppression therapy.
